# A new perspective on the pathophysiology of kidney diseases: the intracellular complement system- “complosome”

**DOI:** 10.3389/fneph.2026.1741099

**Published:** 2026-04-29

**Authors:** Emre Leventoğlu, Sevcan A. Bakkaloğlu

**Affiliations:** 1 Department of Pediatric Nephrology, Konya City Hospital, Konya, Türkiye; 2 Department of Pediatric Nephrology, Faculty of Medicine, Gazi University, Ankara, Türkiye

**Keywords:** complement system, complosome, immunology, kidney disease, nephrology

## Abstract

The complement cascade is a vital component of the innate immune system. It consists of around 50 proteins, including circulating complement proteins and membrane-bound receptors. It operates through three main activation pathways: classical, lectin, and alternative. Dysregulation of the complement system is implicated in several kidney diseases, such as complement-mediated thrombotic microangiopathy, lupus nephritis (LN), C3 glomerulopathy and kidney transplantation. The complement system also extends beyond immune responses, influencing cellular processes through the “complosome” — an intracellular system that regulates metabolism, autophagy, and gene expression. This intracellular complement activity has been linked to kidney diseases, including glomerular and tubular injuries and renal cancer. As research progresses, targeting both extracellular and intracellular complement components holds promise for more effective treatments in complement -driven diseases. In this mini review, the role of the complement system in immune defense, the impact of the alternative pathway in kidney diseases, and the relationship between intracellular complement activity -complosome- and cellular functions have been discussed. Additionally, new approaches for the treatment of complement dysregulation have been explored.

## Physiology of the extracellular complement system

Complement cascade, a key part of the innate immune system, plays a critical role in defending against pathogens like viruses and bacteria. Circulatory and membrane-bound complement proteins interact with immune cells and inflammation mediators, providing effective protection against infections and aiding tissue repair (1).

The complement system comprises around 50 components, including blood- and lymph-circulating proteins, as well as membrane-bound receptors and regulators. These complement proteins act as pattern recognition receptors (PRRs) that detect pathogen-associated molecular patterns (PAMPs) and damage-associated molecular patterns (DAMPs). They operate through a tightly regulated cascade of serine proteases, which can be initiated through three pathways: classical, lectin, and alternative pathways (2). The classical pathway is triggered when C1 complex binds to the Fc region of antigen-bound antibodies, such as IgG or IgM. It is a complex composed of C1q, C1r, and C1s subunits, initiating a proteolytic cascade that cleaves C4 and C2, leading to the formation of C4b2a, the C3 convertase of the classical pathway. This process leads to the generation of C3b, which can bind to cells and facilitate further amplification of complement activation. In addition, the lectin pathway-an antibody independent mechanism-is initiated when lectins such as mannose-binding lectin (MBL) and ficolins bind to microbial surfaces, thereby triggering a sequence of events analogous to those of the classical pathway. The alternative pathway does not need to be triggered by PAMPs. Instead, it is constitutively active to some extent, and continuous C3 cleavage results in C3b generation. Tickover is a unique spontaneous activation mechanism observed in the alternative pathway. In this process, a small portion of circulating C3 undergoes continuous low-level hydrolysis even in the absence of any pathogen. This hydrolyzed C3 creates the liquid phase C3 converter via factors B and D. If this process occurs near a pathogen surface, the produced C3b binds to the surface and triggers the formation of the C3bBb complex, enhancing activation in the pathway through further C3b production (3). The complement system culminates in the formation of the membrane attack complex (MAC), which causes cell lysis by creating pores in the microbial membrane. This process begins with C3 convertases cleaving C3 to produce C3a and C3b, followed by the C5 convertase cleaving C5 into two fragments, C5a and C5b. MAC assembly requires the formed C5b to bind to C6, C7, C8, and C9, respectively. C9 polymerization at the site of the bound C5b-8 complex disrupts the integrity of the membrane, leading to cell death in a manner similar to perforin-mediated lysis by cytotoxic cells (4).

## Multisystem effects of the complement system

**Figure 1:**
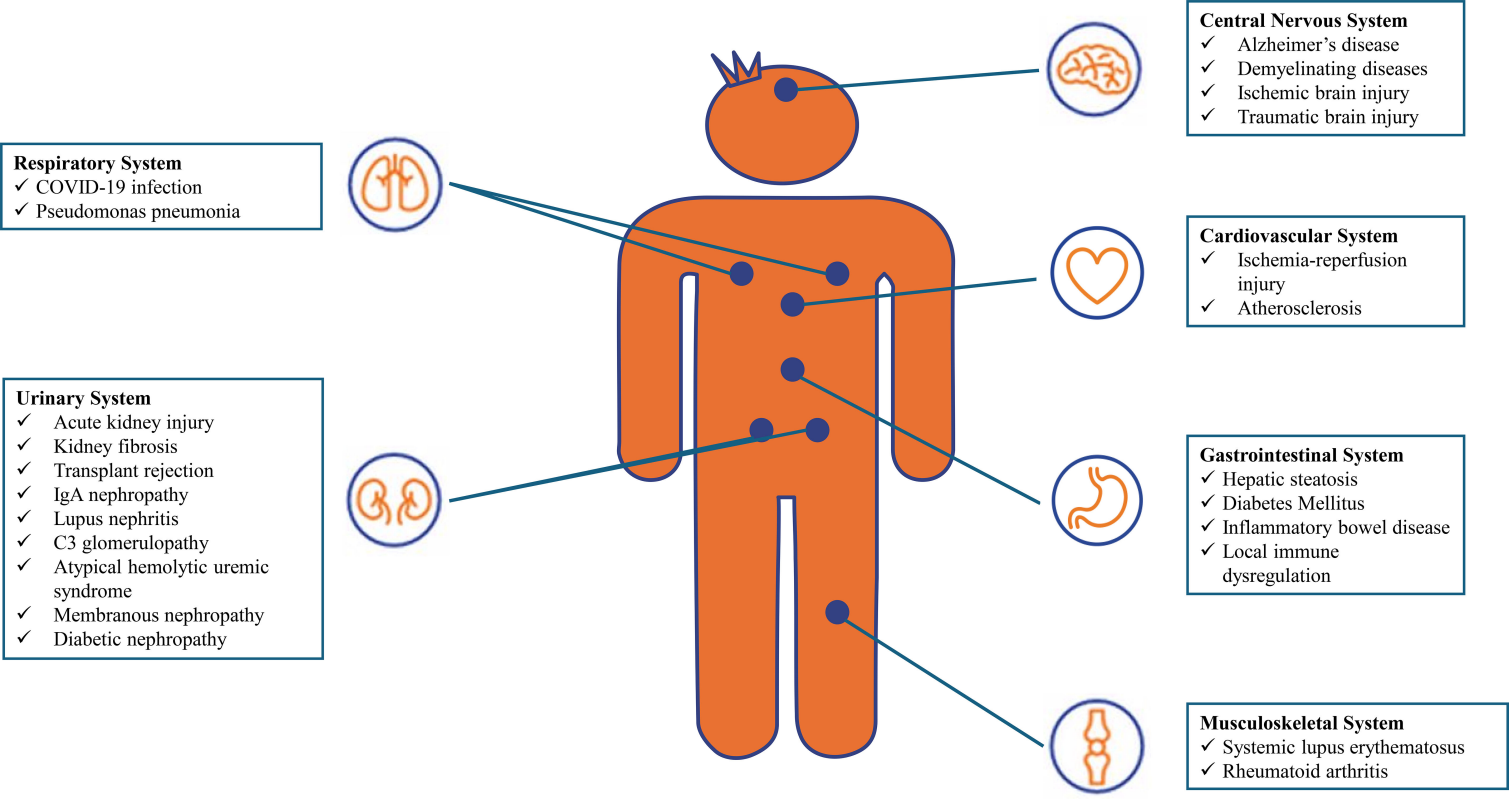
Diseases caused by complement dysregulation in different organ systems.

The complement system is one of the fundamental components of the innate immune response and also plays an important role in maintaining tissue homeostasis. However, excessive or inappropriate activation of this system can lead to inflammatory and degenerative diseases in various organs (**Figure 1**). Among these organs, the kidney is one of the most sensitive to complement dysregulation (5).

The dysregulation of the alternative pathway is a major contributor to several kidney diseases. Excessive activation of complement components and subsequent generation of effector molecules such as anaphylatoxins in kidney can lead to serious tissue damage, including inflammation, thrombosis, and organ dysfunction. This process is particularly relevant in diseases like acute kidney injury (AKI), atypical hemolytic uremic syndrome, C3 glomerulopathy, and immune complex membranoproliferative glomerulonephritis, where complement dysregulation results from genetic or acquired abnormalities (6). In AKI models induced by different stimuli such as ischemia-reperfusion, nephrotoxins, and sepsis, complement activation occurs in the tubulointerstitial area via the alternative pathway. Clinically, increased levels of activation products such as Ba, Bb, C3a, and C4a in urine and plasma in both adult and pediatric AKI indicate the contribution of complement to the course of the disease (7). In addition, while the classical pathway is triggered by immune complex deposition in IgA nephropathy, the alternative and lectin pathways play a critical role in the inflammation process (8). Although lupus nephritis (LN) activates the complement system primarily via the classical pathway (triggered by C1q binding to immune complexes) through immune complexes deposited in the kidney, increased plasma level of Bb has been demonstrated in active LN patients; this finding suggests that activation of the alternative pathway also plays a role (9). Lower levels of C1q and C3 in these patients may predict poor outcomes, including disease progression. Patients with C3 deposition without C1q in their biopsies tend to have worse responses to immunosuppressive treatments and more rapid progression of kidney damage (9). Kidney transplantation offers better survival and quality of life compared to dialysis, but complications such as ischemia-reperfusion injury (IRI), delayed graft function (DGF), T-cell and B-cell mediated rejection, and chronic allograft injury can negatively impact long-term graft survival. During IRI, the complement system is primarily activated via the lectin pathway, leading to inflammation, cell damage, and fibrosis. Increased complement activation, particularly elevated C5b-9 and C3d levels, is associated with DGF and poor graft outcomes. Complement activation also contributes to T-cell priming and antibody-mediated rejection (AMR), where donor-specific antibodies trigger the classical pathway, leading to graft injury (10). Therefore, complement system plays a critical role in these processes, making it a potential target for therapeutic strategies aimed at improving graft survival and minimizing long-term complications such as fibrosis and chronic rejection (10). The introduction of eculizumab into clinical practice has demonstrated that complement inhibitors can improve the natural course of complement-mediated kidney diseases. Following this development, some molecules (ravulizumab, crovalimab, avacopan, danicopan, iptacopan, pegcetacoplan, narsoplimab) have been developed in both adult and pediatric nephrology. Many of these agents have yielded improved outcomes, such as reduced proteinuria and preserved kidney function (11).

## Intracellular complement system: “complosome”

Although the complement system is traditionally thought to function in the circulation and extracellular environment, the system also has an intracellular component (12). This intracellular complement system, called the “complosome,” is not only part of the immune response but also a regulator of cellular metabolism and physiology (3). Basic complement proteins such as C3 and C5, along with their activation products, receptors, and regulators, interact with intracellular danger sensors and effector systems such as inflammasomes, autophagosomes, and ribosomal mechanisms that help regulate cell metabolism, autophagy, and gene expression (13, 14).

**Figure 2:**
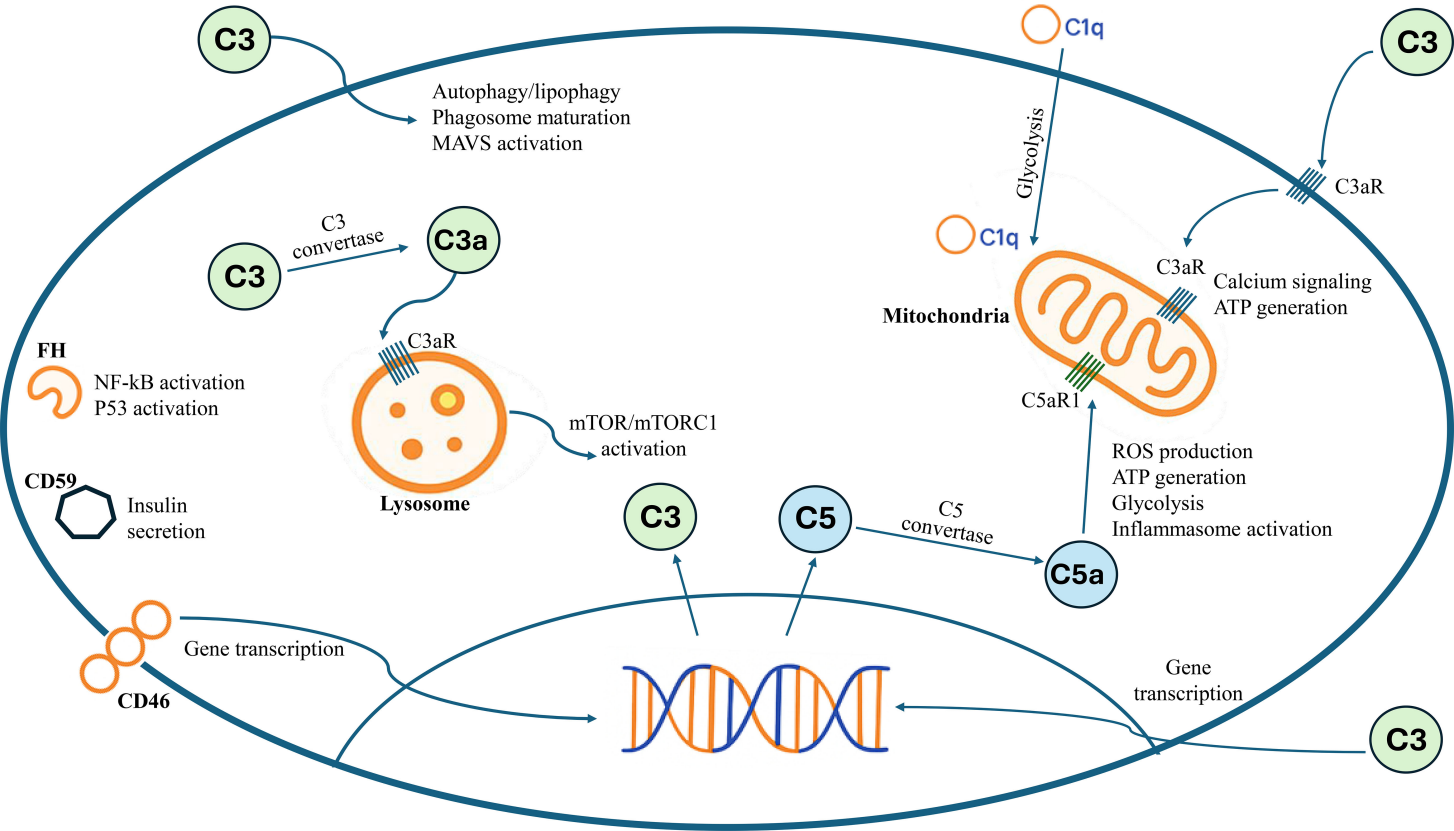
The intracellular complement system and its functions in cell metabolism.The complosome consists of intracellular components found in various immune and non-immune cells. Molecules produced by the cleavage of C3 and C5 within the cell play a role in regulating mTOR signaling, energy metabolism, and inflammatory responses via lysosomal and mitochondrial receptors. NF-Kb, Nuclear Factor kappa B; ATP, Adenosine triphosphate; ROS, Reactive oxygen species.

Complosome components have been found in various cellular compartments, including the cytoplasm, nucleus, lysosomes, endoplasmic reticulum, and mitochondria (15). Interestingly, C3 and C5 can be activated by specific proteases or C3/C5 convertases on the plasma membrane or within subcellular compartments. Components of the complosome can undergo post-translational modifications and exhibit different structures based on the cell’s environment. In CD4+ T cells, lysosomal C3 is cleaved by cathepsin L, which activates the mTOR signaling pathway, promoting cell survival. The CD46 receptor plays a key role in this process by preventing Notch-1 activation in resting cells, and when stimulated, it can regulate T cell responses, including the release of interferon-gamma (IFN-γ) and differentiation into Th1 cells (16). Additionally, CD46 promotes glycolysis and oxidative phosphorylation, supporting the metabolic needs of activated T cells. The complosome also regulates mitochondrial oxygen metabolism through C5aR1, influencing cellular energy production and inflammasome activation (16). Unlike C5aR1, C5aR2 is thought to be largely localized in intracellular vesicles and generally functions as a ‘scavenger’ receptor that dampens the C5a signal. However, the potential signaling roles of intracellular C5aR2 remain an area that has yet to be fully explored (17). The intracellular complement system is shown in **Figure 2**.

Tubular epithelial cells in the kidney are the primary source of intrarenal complement production and can synthesize nearly all complement proteins under both physiological and pathophysiological conditions (18). Proximal tubular epithelial cells in particular produce C3, and this production increases in the presence of proinflammatory cytokines such as IL-1, IL-2, IFN-γ, and IL-17. The production of Factor B, C2, C4, and Factor H also increases when stimulated by IFN-γ and IL-1. On the other hand, the anti-inflammatory cytokine TGF-β suppresses complement production in proximal tubular epithelial cells (19). Glomerular mesangial and epithelial cells, as well as podocytes, also produce various complement and regulatory proteins. In culture conditions, expressions of C1q, C1r, C2, C3, C3aR, C5aR, C7, CR1, and CR2 have been detected in podocytes, and this expression increases due to podocyte injury (20). In terms of intracellular mechanisms, it is thought that C3 and C5 can be activated in lysosomes and other subcellular compartments via specific proteases or convertases in kidney cells. This activation triggers cellular signaling pathways such as mTORC1 pathways, mitochondrial reactive oxygen radicals’ production, and inflammasome activation. These data reveal that intracellular complement activation in kidney cells involves a complex mechanism related to both damage and regulatory signals, and that pathogenic effects may vary depending on the cellular context (21).

Dysregulation of the intracellular complement system has been associated with many kidney diseases. Historical studies conducted in the 1990s showed that the kidney is not only a target organ of the complement system, but also an active complement production site (18, 22–25). Although the liver is the primary source of complement proteins in circulation, high C4 gene expression has also been reported in renal epithelium; this suggests that the kidney may contribute to immune defense by locally producing complement components (22). While C3 expression is limited in normal kidney tissue, it increases significantly in diseases such as LN and focal segmental glomerulosclerosis (23). Indeed, it has been shown that C3 and C4 expressions, which can be synthesized by glomerular and tubular epithelial cells, particularly increase when stimulated by IFN-γ (24, 25). In an Adriamycin-induced proteinuria model by Sheerin NS et al., kidney grafts lacking local C3 production were found to be protected from complement activation, tubular injury, and progressive kidney dysfunction, despite the presence of abundant circulating C3. These findings suggest that local complement activation underlies proteinuria-related kidney damage and may be a common pathogenic mechanism in many immunological or non-immunological kidney diseases (26). Locally produced complement components also play important roles in kidney transplantation. A study by Serinsöz E et al. showed that local C3 production in tubular epithelial cells increased during both humoral and cellular rejection in renal allografts, and this was particularly evident in C4d-positive and T-cell-mediated rejection cases (27). Therefore, kidney epithelial cells appear to play an active role in both physiological defense and pathological inflammatory responses through local IFN-γ production.

## New hopes for treatment of kidney diseases: targeting the complosome

The complement signature in kidney disease is new research of interest. LN and C3 glomerulopthy have now been considered potential complosome-driven diseases. Variations in complosome activity have been linked to tissue damage and disease progression in kidney cells. In glomerular endothelial cells, the absence of Factor H, a regulatory protein, results in cytoskeletal remodeling, loss of barrier function, and increased cell proliferation, contributing to pathological angiogenesis (14). The complosome also affects the physiology of tubular cells. Single-cell RNA sequencing has identified elevated levels of C3 and C5 in tubular cells following injury, indicating a key role for the complosome in kidney responses to stress (28). Intracellular C5 and C5aR1 in kidney macrophages are involved in folic acid-induced tubular injury, and C5aR1 deficiency can reduce renal fibrosis (29).

A recent study identified five distinct patient groups with different phenotypic and complement profiles in C3G and IC-MPGN patients using hierarchical clustering analysis. Notably, in one cluster, despite normal systemic complement activation, there was a high accumulation of glomerular C3, suggesting solid-phase complement activation. This finding suggests that locally initiated complement activation in some patients may lead to kidney damage without causing significant changes in systemic levels. These patients also had the highest incidence of renal failure during follow-up, indicating that solid-phase activation may have a significant impact on clinical prognosis (30).

Cell-permeable complement inhibitors have reduced inflammation by suppressing intracellular complement overactivation in various diseases. Cell-permeable inhibitors targeting cathepsin L, factor B, and C5aR1 have reduced IFN-γ production, SARS-CoV-2-triggered cellular C3 activation, and proinflammatory responses such as IL-1β in macrophages/monocytes (29, 31, 32). Therefore, we believe that the discovery of cell-permeable complement blockers may offer new therapeutic hope for patients with conditions such as C3 glomerulopathy who do not respond adequately to extracellular complement blockade.

## Challenges and limitations in complosome

Despite recent developments, the molecular mechanisms and regulatory factors of complosome activity have not yet been fully elucidated. However, these methodological challenges have important implications for the interpretation of complosome data. The distinction between intracellular and extracellular complement systems is methodologically difficult, raising concerns about whether observed effects truly reflect autonomous complement activation within cell rather than contamination from extracellular sources. Therefore, the limited availability of techniques that enable precise subcellular targeting further complicates efforts to characterize the dynamics of complosome signaling (13).

Animal models present additional challenges. The absence of CD46 in somatic tissues in rodents and the controversial expression of C3aR and C5aR in mouse T cells limit the usability of animal models. Consequently, findings derived from rodent studies must be interpreted cautiously. Furthermore, since cell-autonomous C3 differs structurally and post-translationally from liver-derived C3, specific reagents are needed to distinguish this difference (33). This limitation may increase the risk of misclassification and may lead to over- or underestimation of complosome activity in experimental systems.

Considering these challenges, complosome-related findings should be interpreted within the context of these limitations, and future studies will require additional tools to further define intracellular complement functions.

## Conclusion

The complement dysregulation, including abnormal complosome activity, contributes to a range of prevalent human diseases. However, current complement-targeting therapies mainly address extracellular complement components. Given the emerging role of the complosome in controlling basic cellular functions in both immune and non-immune cells, targeting intracellular complements, potentially alongside traditional extracellular therapies, could offer a broader and more effective approach. Recent studies have demonstrated the potential of cell-permeable inhibitors, such as CTSL and FB inhibitors, in modulating intracellular complement activity and reducing inflammation. Individually designed anti-complement therapeutics based on the unique complotype of the patient might be a future treatment prospect for the patients.
